# Booster-free anti-retroviral therapy for persons living with HIV and multidrug resistance (B-Free): protocol for a multicentre, multistage, randomised, controlled, non-inferiority trial

**DOI:** 10.1136/bmjopen-2024-094912

**Published:** 2024-11-21

**Authors:** Marie Ballif, Dominique Braun, Alexandra Calmy, Enos Bernasconi, Matthias Cavassini, Frédéric Tissot, Marcel Stoeckle, Patrick Schmid, Christoph A Fux, Marc Van der Valk, Kees Brinkman, Tania Mudrikova, Fabrice Bonnet, Olivier Leleux, Manuela Saúde, Daniela Hirter, Nathalie Schwab, Andreas Limacher, Felix Rintelen, Roger Kouyos, David Haerry, Sofia C. Zambrano, Martina Egloff, Christina Akre, Isabelle Peytremann-Bridevaux, Andri Rauch, Gilles Wandeler, Bernard Surial

**Affiliations:** 1 Department of Infectious Diseases, Inselspital, Bern University Hospital, University of Bern, Bern, Switzerland; 2 Institute of Social and Preventive Medicine (ISPM), University of Bern, Bern, Switzerland; 3 Department of Infectious Diseases and Hospital Epidemiology, University Hospital Zurich, Zurich, Switzerland; 4 HIV Unit, Division of Infectious Diseases, Geneva University Hospitals, Geneva, Switzerland; 5 Division of Infectious Diseases, Ente Ospedaliero Cantonale Lugano, University of Geneva and University of Southern Switzerland, Lugano, Switzerland; 6 Division of Infectious Diseases, University Hospital of Lausanne, University of Lausanne, Lausanne, Switzerland; 7 Division of Infectious Diseases and Hospital Epidemiology, University Hospital Basel, University of Basel, Basel, Switzerland; 8 Division of Infectious Diseases, Infection Prevention and Travel Medicine, Cantonal Hospital of St Gallen, St Gallen, Switzerland; 9 Division of Infectious Diseases, Cantonal Hospital of Aarau, Aarau, Switzerland, Aarau, Switzerland; 10 HIV Monitoring Foundation, Amsterdam, Netherlands; 11 Amsterdam UMC, University of Amsterdam, Department of Infectious Diseases and Amsterdam Institute for Immunology & Infectious diseases, Amsterdam, Netherlands; 12 Department of Internal Medicine, OLVG, Amsterdam, Netherlands; 13 Department of Internal Medicine and Infectious Diseases, University Medical Center Utrecht, 3584 CX, Utrecht, Netherlands; 14 University of Bordeaux, INSERM, Institut Bergonié, BPH, Bordeaux, France; 15 CHU Bordeaux, Hôpital Saint-André, Service de Médecine Interne et Maladies Infectieuses, Bordeaux, France; 16 Department of Clinical Research, University of Bern, Bern, Switzerland; 17 Institute of Medical Virology, University of Zurich, Zurich, Switzerland; 18 Chair Positive Council, Zürich, Switzerland; 19 Centre for Primary Care and Public Health (Unisanté), University of Lausanne, Lausanne, Switzerland

**Keywords:** HIV & AIDS, Randomized Controlled Trial, Protocols & guidelines

## Abstract

**Introduction:**

Anti-retroviral therapy (ART) simplification strategies are needed for treatment-experienced people with HIV (PWH) and multidrug-resistant viruses. These individuals are commonly treated with boosted ART regimens and are thereby at risk for harmful drug-drug interactions (DDI). In this trial, we aim to assess the efficacy of the combination doravirine, dolutegravir and lamivudine (DOR/DTG/3TC) among people with a history of virological failure who receive boosted ART.

**Methods and analysis:**

B-Free is a multistage, randomised, multicentre, open-label, non-inferiority trial, embedded within the Swiss HIV Cohort Study and conducted in collaboration with cohorts of PWH in the Netherlands and France. Cohort participants with a history of ART change due to virologic failure and who maintain HIV virologic suppression with an ART regimen consisting of a pharmacological booster and at least two drugs from classes other than nucleoside reverse transcriptase inhibitors are included. Patients with major drug resistance mutations against DTG or DOR and individuals with chronic hepatitis B virus infection are not eligible for the study. Individuals are randomised 1:1 to either receiving co-formulated DTG/3TC and DOR once daily or continuing their boosted ART regimen. The primary outcome is the proportion of individuals lacking virologic control (HIV-RNA ≥50 cp/mL) at 48 weeks, according to the Food and Drug Administration snapshot algorithm. Changes in DDI burden (assessed using a DDI score), treatment satisfaction (assessed using the HIV Treatment Satisfaction Questionnaire), quality of life and mental health represent key secondary outcomes. Additional secondary outcomes include the proportion of individuals developing new resistance-associated mutations and changes in quality of life and mental health. In a qualitative substudy, we will conduct semistructured interviews with a subset of participants to assess their expectations and experiences towards HIV treatment and clinical research in general. Enrolling 210 individuals will provide 80% power to demonstrate non-inferiority, defined as less than 8% absolute increase in loss of viral suppression in individuals randomised to DOR/DTG/3TC (one-sided type I error rate of 0.025).

**Ethics and dissemination:**

The study was approved by the competent ethics committees (reference number BASEC 2023–01060) and the regulatory authority Swissmedic (reference number 701655) in Switzerland before the enrolment of the first participant. Approval by the European Medicines Agency and local ethical committees in the Netherlands and France will be obtained prior to including participants in these countries. Participant’s written informed consent is obtained by the investigators before enrolment. The results of all major B-Free study outcomes will be submitted to peer-reviewed journals that enable Open Access publication.

**Trial registration number:**

Swiss National Clinical Trials Portal (SNCTP000005686, registered on 06 November 2023) and Clinicaltrials.gov (NCT06037564, registered on 07 September 2023).

**STRENGTHS AND LIMITATIONS OF THIS STUDY**

- B-Free will evaluate simplified anti-retroviral treatment strategies for persons with a history of treatment failure.
- The study aims at improving HIV treatment in an ageing population with a high burden of comorbidities and a high risk for experiencing drug-drug interactions, which is a pressing clinical concern.
- The study population will be recruited within established cohorts of people with HIV (PWH), facilitating participant identification and recruitment and guaranteeing long-term outcome assessment after the end of the study.
- The strong collaboration with patient representatives helped us design the study according to priorities and perceptions of PWH, and the integration of a strong qualitative part into our work allows us to shape and optimise our multistage trial prospectively.
- Due to the heterogeneous anti-retroviral treatments used in the control arm, neither participants nor study physicians can be blinded to the treatment allocation, but all laboratory assessments and statistical analyses of the primary outcome are performed by individuals blinded to the treatment allocation.

## Introduction

 Multiple anti-retroviral therapy (ART) simplification strategies exist for people with HIV (PWH), including dual therapies and long-acting injectable ART. Strong evidence from clinical trials has confirmed the efficacy and safety of these simplified regimens.^1^,^3^ However, these simplification trials were mainly limited to individuals with uncomplicated HIV infection, and only very few clinical trials have assessed ART optimisation strategies for PWH and a history of virological failure.^4^

Evidence on treatment simplification strategies for individuals with a long-standing HIV infection and acquired resistance is needed. Because of the high barrier to resistance of boosted protease inhibitors, this population commonly receives boosted regimens, increasing their risk of experiencing drug-drug interactions (DDI) with co-medications used to treat comorbidities.^5^ New drugs, including late generation non-nucleoside reverse transcriptase inhibitors (NNRTI) and integrase strand transfer inhibitors (INSTI) have a lower potential for DDI while retaining a high barrier to resistance.^6 7^ Combining these newer substances may provide simplification strategies for treatment-experienced PWH and multidrug resistance.

‘Booster-Free antiretroviral therapy for persons living with HIV and multidrug resistance’ (B-Free) is a multistage trial to evaluate ART optimisation strategies among individuals with a history of virological failure. B-Free is embedded within the Swiss HIV Cohort Study (SHCS) and is conducted in collaboration with HIV cohorts in the Netherlands and in France. In its first stage, we will assess the efficacy and safety of combining doravirine, dolutegravir and lamivudine (DOR/DTG/3TC) compared with continuing boosted ART in individuals with multidrug-resistant HIV.

## Methods and analysis

### Design and setting

**Figure 1:**
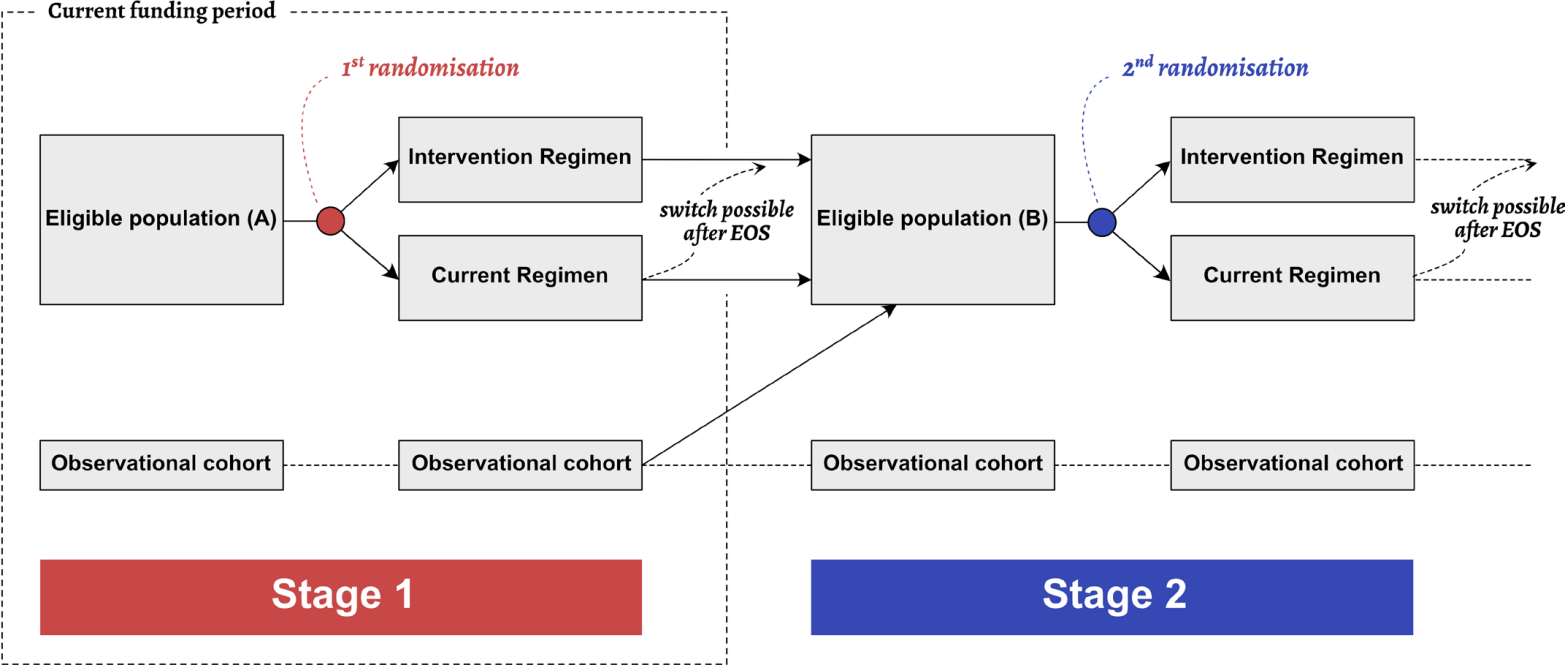
The multistage trial framework of B-Free. At each trial stage, eligible participants are randomised into one of two arms. People who are not eligible for stage 1 will continue follow-up in their respective HIV cohort and will be re-assessed for eligibility at a later trial stage. The process can be repeated when new HIV drugs are marketed (here exemplified as stage 2). This protocol describes the stage 1 trial. EOS, end of study.

B-Free is a multistage, randomised, multicentre, open-label, non-inferiority trial, which is embedded within the SHCS and cohorts in the Netherlands and France.^8^,^10^ The multistage design is adapted from the ‘Multi-arm multi-stage platform design’ framework.^11 12^ At each stage, individuals who fulfil the eligibility criteria will be randomly assigned 1:1 to either receive the new booster-free intervention regimen or to remain on their current treatment (figure 1).

This trial protocol describes the first stage of the multistage trial. In this stage, we aim to include 210 PWH-1 infection who had a history of ART change due to virologic failure and have stable HIV suppression on ART including a pharmacological booster (ritonavir or cobicistat) and at least two agents from the NNRTI, protease inhibitor (PI) or INSTI classes. Eligible study participants will be randomly assigned 1:1 to one of the two following study arms:

- *Booster-free intervention arm*: participants are switched to oral DOR 100 mg and co-formulated DTG 50 mg and 3TC 300 mg. This two-pill regimen is taken, independent of meals.
- *Control arm*: participants randomised to this arm continue their current oral booster–containing ART regimen. The dosing schedule remains unchanged. To minimise spillover effects, changes in ART regimens are discouraged and limited to occurrence of virologic failure, new DDIs or the onset of ART-related adverse events (AEs) requiring modification.

Treatment-experienced individuals who are not eligible to receive the current intervention regimen (‘observational cohort’) are identified and followed within the cohort. They will be reassessed for participation at a later trial stage.

Once the first stage is completed and new drugs or ART combinations become available, a new stage will be planned, and eligibility criteria adapted accordingly. For the next trial stage, all B-Free participants as well as individuals from the observational cohort will be re-assessed for eligibility.

### Objectives and study outcomes

The primary objective is to evaluate whether DOR/DTG/3TC given one time per day is non-inferior to a boosted ART regimen in maintaining HIV suppression among PWH and previous virologic failure with a non-inferiority margin of 8 percentage points. The secondary objective is to determine whether switching to DOR/DTG/3TC leads to a lower burden of DDI and better treatment satisfaction compared with continuing booster-containing ART. The study outcomes are outlined in box 1.

Box 1 **Primary, secondary and safety outcomes of B-Free**

**Primary outcome**

- Difference in the proportion of individuals with an HIV-RNA ≥50 cp/mL at 48 weeks between the two treatment arms (as recommended by the FDA snapshot approach for trials assessing ART switches).

**Key secondary outcomes**

- Changes in the burden of DDI from weeks 0 to 48.
- Changes in treatment satisfaction between weeks 0 and 48.

**Further secondary outcomes**

- Proportion of patients experiencing confirmed virologic failure, defined as two consecutive HIV-RNA measurements ≥200 cp/mL.
- Proportion of individuals experiencing impairment or loss of future drug options, defined as new detection of resistance-associated mutations against DOR, DTG and 3TC (intervention-arm) or against the components of the ART regimen that the virus was considered to be sensitive to at randomisation (control-arm).
- Proportion of individuals with any moderate or severe DDI at any study visit.
- Proportion of patients for which the treating physician would have liked to prescribe a drug but abstained from it due to DDI issues with the ART.
- Differences in quality of life between both groups at week 48.
- New onset of depression.
- Changes in intact proviral HIV-DNA levels in PBMC.
- Proportion of individuals with an ‘anti-HBc alone’ who develop a detectable hepatitis B viral load.
- Cumulative cost of all ART drugs used.

**Other safety outcomes**

- Safety outcomes include changes in CD4 cell count, blood lipid values, body weight, body mass index, renal and liver function, and onset of new drug-related central nervous system AEs, and SAEs.

ART, anti-retroviral therapy; DDI, drug-drug interaction; FDA, Food and Drug Administration; PBMC, peripheral blood mononuclear cell; SAE, serious adverse event.

### Study population and recruitment

We include treatment-experienced individuals with a history of virological failure and who currently receive a complex ART regimen which includes a booster. Detailed eligibility criteria are provided in box 2. The established cohort infrastructures greatly facilitate the recruitment of trial participants. Clinical and laboratory data are available to assess trial eligibility. Potential participants are seen every 3 to 6 months in the participating centres or can be contacted by the treating physicians between study visits. In the trial preparation phase, 328 eligible individuals were identified in the SHCS and 171 individuals who were followed in two clinics in Amsterdam. In a preliminary survey among 121 potentially eligible SHCS participants, 88 (72%) responded that they were interested in participating in a study aiming at evaluating novel HIV therapy combinations with a reduced risk of DDI. Assuming a more conservative participation rate of 50% of eligible patients, we expect to reach the recruitment targets within 2 years.

Box 2 **Inclusion and exclusion criteria for B-Free stage 1**

**Inclusion criteria**

- Informed consent as documented by signature.
- Age ≥18 years.
- Documented HIV-1 infection.
- On ART including a pharmacological booster (ritonavir or cobicistat) and at least 2 drugs from classes other than NRTI.
- A history of ART change due to virologic failure.
- HIV-RNA <50 cp/mL at screening and for at least 24 weeks before screening (one blip with less than 200 cp/mL is allowed).

**Exclusion criteria**

- Creatinine clearance <30 mL/min.
- Known hypersensitivity, allergy or intolerance to DOR, DTG or 3TC.
- Presence of major drug resistance mutations against DTG (G118R, G140R, Q148H, Q148K, Q148R, R263K) or DOR (V106A, Y188L, F227C, F227L, M230L, Y318F) according to IASUSA in individual cumulative resistance analyses*.
- Concomitant use of drugs that decrease DTG or DOR blood concentrations.
- Chronic hepatitis B infection.
- Women who are pregnant or breastfeeding.
- Concurrent participation in another ART intervention study.

*Persons without available resistance testing will not be excluded if no resistance to dolutegravir or doravirine is assumed based on ART history. 3TC = lamivudine; ART, antiretroviral therapy; DOR, doravirine; DTG, dolutegravir; NRTI, nucleostide reverse transcriptase inhibitor.

### Qualitative substudy

We undertake semistructured interviews to evaluate the acceptability of participating in an interventional ART trial, the needs and expectations concerning booster-free regimens or ART in general, and to evaluate how the needs of persons living with HIV can best be addressed by research. These interviews are conducted among 30 trial participants (15 in the intervention and 15 in the control arm). Interviews are done at baseline and after 1 year to identify any changes in perception or expectations resulting from their participation in the trial. In addition, similar interviews are conducted among individuals who were ineligible for the trial (n=15), or who declined participation (n=15). These individuals are only interviewed once, as no major change is expected to occur over time.

## Randomisation

Randomisation is stratified by participating centres. Participants are randomised 1:1 using randomly permuted blocks of varying sizes to one of the study arms, using a web-based randomisation system. Access to the randomisation list is restricted to an individual who is not involved in trial-related tasks, and allocation concealment ensured, as the system only releases the treatment allocation at the time of randomisation.

### Intervention and blinding

**Figure 2:**
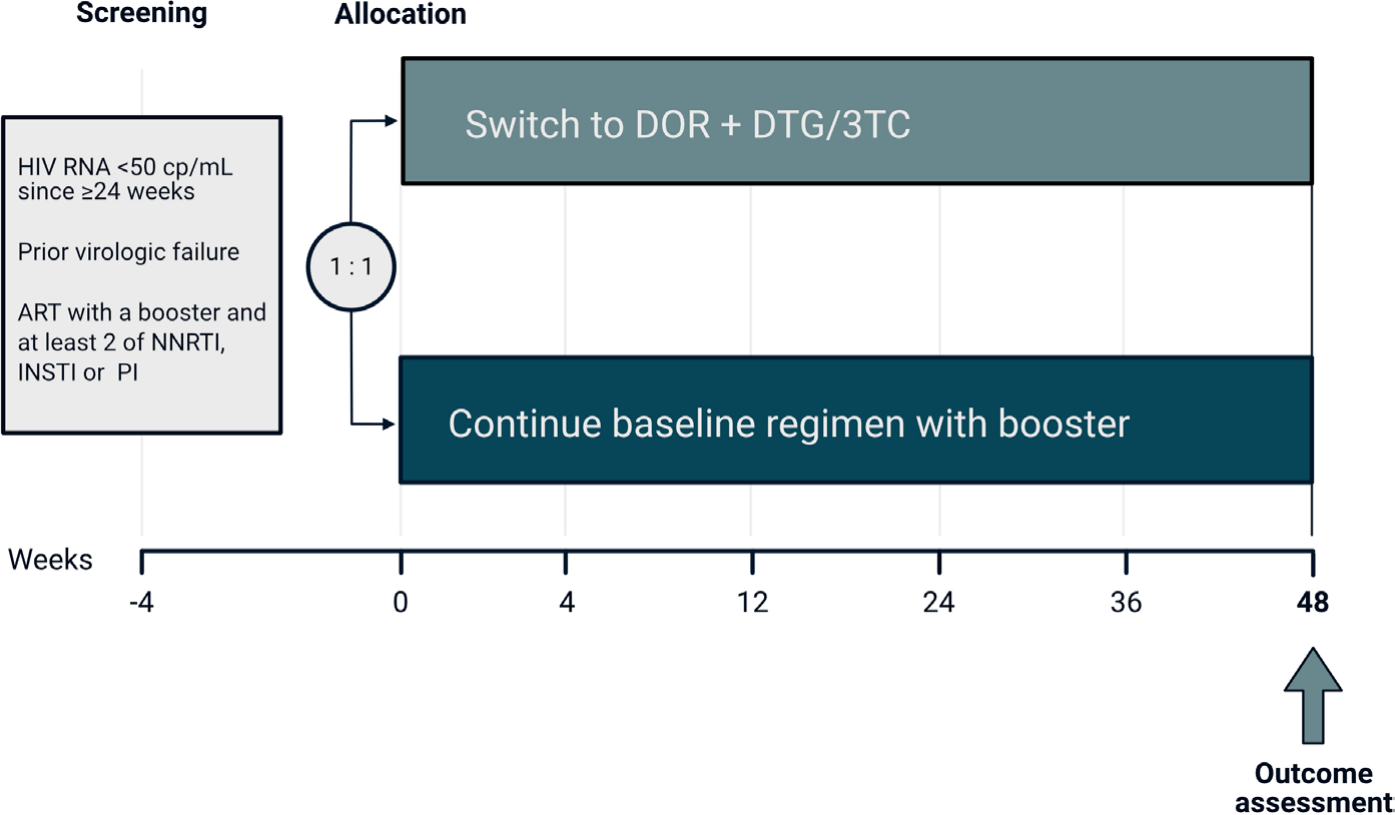
Flow of study participants included in the first stage of the B-Free trial. 3TC, lamivudine; ART, anti-retroviral therapy; DOR, doravirine; DTG, dolutegravir; INSTI, integrase strand transfer inhibitor; NNRTI, non-nucleoside reverse transcriptase inhibitor; PI, protease inhibitor.

Participants are randomised to receive DOR 100 mg and co-formulated DTG/3TC 50/300 mg one time per day (intervention) or to continue their current and fully suppressive ART (control, figure 2). The study drugs are dispensed by the study site or local pharmacy at each study visit. Since ART regimens in the intervention and the control arm may differ substantially in the number of pills, blinding of treatment allocation was deemed to be impractical. However, virologic outcome assessment in the laboratory is blinded, and treatment assignments will be masked from the trial statistician for the analyses of the primary and the main secondary outcomes.

### Assessment of primary outcome

Study assessments and the study schedule of the B-Free trial are summarised in online supplemental table S1. The primary outcome is ‘lack of HIV viral suppression’, defined as an HIV viral load of ≥50 cp/mL at week 48 (time window ±14 days, box 2). HIV-1 RNA levels in plasma will be quantified by PCR in local accredited laboratories. In case of technical problems or an HIV viral load ≥50 cp/mL, the study participant is asked to return for a repeat measurement as soon as possible. No more than one HIV viral load re-test is allowed. Individuals who prematurely discontinue the study will be included in the proportion of participants with a lack of viral suppression if their last HIV viral load was ≥50 cp/mL. Individuals without available data at 48 weeks and the last HIV viral load <50 cp/mL will be categorised into one of the following categories: ‘discontinued due to AE/death’, ‘discontinued for other reasons’ or ‘on study but missing data during analysis window’.^13^

### Assessment of key secondary outcomes

*DDI*: he burden of DDI is assessed using a DDI score calculated based on the categories of the University of Liverpool Drug Database. Prescribed ART and co-medications are categorised as *red flag* (3 points) when co-administration is contraindicated, *amber flag* (2 points) for DDIs manageable by dose adjustment or monitoring, *yellow flag* (1 point) for DDIs with no need of a priori dosage adjustment or monitoring, and *green flag* (0 points) for no interaction.^14^ The DDI score represents the sum of all points and is assessed at baseline and week 48.

*Treatment satisfaction and other patient-reported outcomes:* validated instruments are used to assess treatment satisfaction, quality of life and mental health at baseline and week 48 (table 1).

**Table 1:** Instruments to evaluate patient-reported outcomes

Dimension	Description
Treatment satisfaction	
HIV treatment satisfaction questionnaire (status version, HIVTSQ)^*^	HIV-specific measure for treatment satisfaction.
HIV treatment satisfaction questionnaire change version (HIVTSQc)^*^	Based on HIVTSQ, but more sensitive to changes over time.
Quality of life	
World Health Organization Quality of Life Brief Version (WHOQOL-HIV BREF)^†^	Instrument to measure quality of life, including HIV-specific questions.
Mental health	
Patient Health Questionnaire (PHQ-9)^‡^	Depression screening instrument.

* Woodcock *et al*. validation of the revised 10-item HIV Treatment Satisfaction Questionnaire status version and new change version. *Value Health*. Sep-Oct 2006;9 (5):320–33.

† WHO. WHOQOL-HIV BREF 2012 revision. https://apps.who.int/iris/handle/10665/77775 (accessed 04.04.2023)

‡ Kroenke *et al*. The PHQ-9: Validity of a brief depression severity measure. *J Gen Intern Med*. Sep 2001;16(9):606–13.

### Managing detectable HIV viral loads during the study

**Figure 3:**
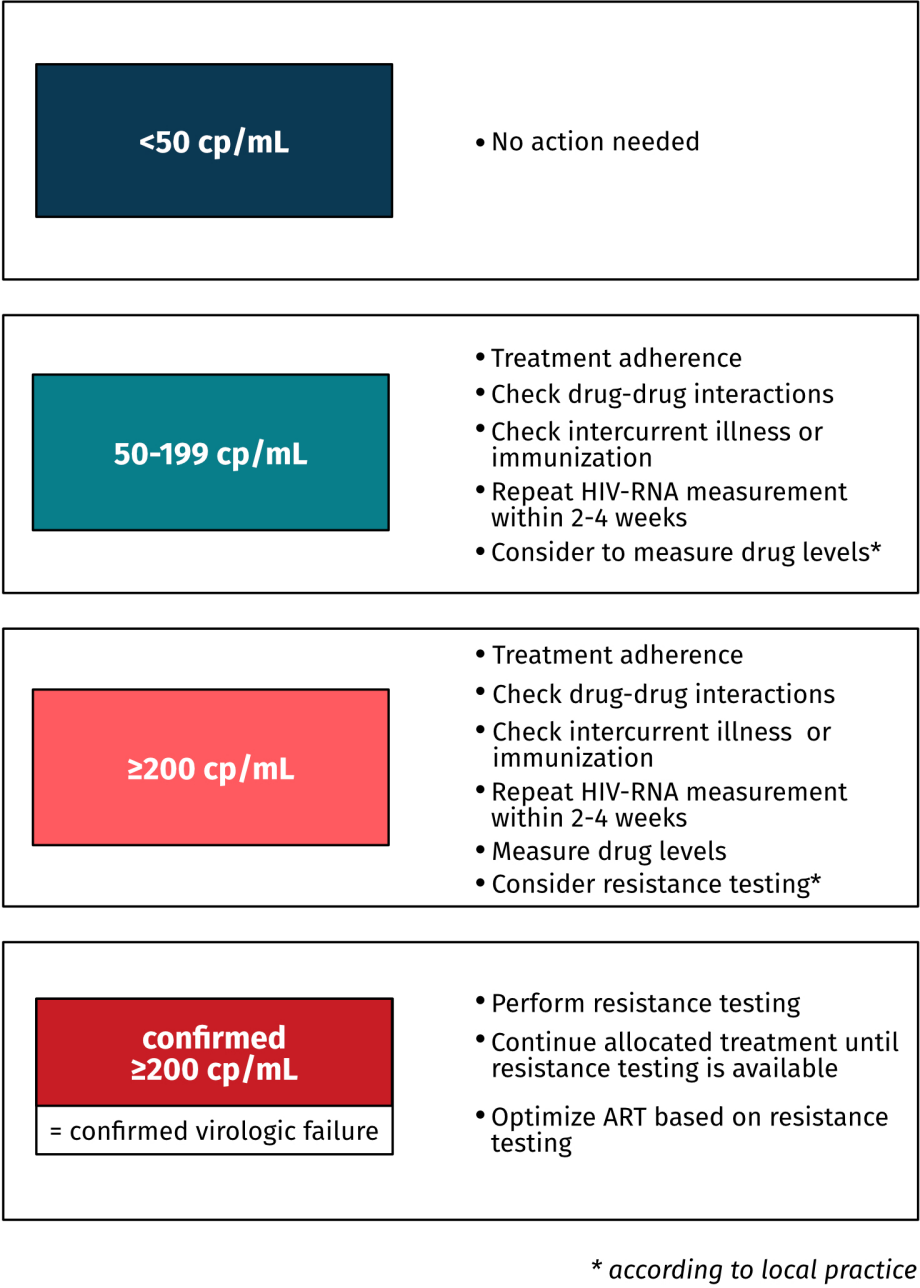
Guidance for viral load monitoring and further assessment if HIV-RNA is detectable. ART, anti-retroviral therapy.

To ensure equal treatment of individuals in both trial arms and across all study sites, a sequence of steps needs to be followed in participants with detectable HIV viral loads during the study. In addition to repeating the HIV-RNA measurement within 2–4 weeks, the steps include assessing treatment adherence, asking for new or inadvertent use of substances that may interfere with the ART regimen, evaluating whether intercurrent illnesses or recent immunisations occurred, measuring plasma drug concentrations and genotypic resistance testing (figure 3). For patients with confirmed virologic failure (HIV-RNA ≥200 cp/mL in two consecutive measurements), genotypic resistance testing is performed. While waiting for the genotypic resistance testing to return, individuals continue their allocated treatment. Participants will receive an individually optimised anti-retroviral treatment based on the results of genotypic resistance testing and will continue to be assessed for follow-up until week 48.

### Statistical analysis

#### Sample size calculation

For the first trial stage, the sample size was calculated to evaluate the non-inferiority of the primary outcome loss of virologic suppression (proportion of individuals with HIV-RNA ≥50 cp/mL) at week 48. The sample size was calculated using the following assumptions:

- *Proportion with loss of viral suppression at48weeks*: in a previous switch trial conducted within the SHCS (Simpl’HIV study), 2.2% of individuals had an HIV viral load ≥50 cp/mL after 1 year.^15^ However, individuals considered for the Simpl’HIV study generally did not have viruses with drug-resistance mutations, whereas in the present study, individuals will be required to have had a history of switching treatment due to lack of efficacy. In this second-line setting, we therefore assume the failure rate to be 4%.
- *Non-inferiority margin*: we set the non-inferiority margin at 8 percentage points. Given that we assume that 4% of individuals will have a detectable HIV viral load at 48 weeks, such a non-inferiority margin would consider 12% of individuals with a detectable HIV viral load to be acceptable with the new treatment. Such a threshold is clinically acceptable for a patient population with long-standing HIV infection and acquired resistance mutations, given the potentially large benefits of the new treatment (lower pill count, improved safety regarding DDIs and possibly better tolerability).

Given the assumptions above, we need to include 190 individuals (95 in each arm) to have 80% power to show non-inferiority at a one-sided alpha level of 0.025. We accounted for an attrition of 10% of individuals during the study and therefore aim to include 210 individuals. This sample size will provide adequate power for both the intention-to-treat (ITT) and the per protocol analysis.

### Analysis populations

We will test for non-inferiority using two analysis populations: (1) the ITT population including all individuals as randomised, irrespective of whether they received the treatment or not, and (2) the ‘per protocol’ (PP) population. Participants will be excluded from the PP population if they did not meet relevant eligibility criteria, did not start their assigned study treatment, discontinued the study treatment prematurely for other reasons than virologic failure and took less than 80% of ART doses throughout the study period.

### Statistical approach

For the primary outcome *loss of HIV viral suppression*, the intervention regimen will be compared against the current regimen at 48 weeks in the ITT and PP participant set. A risk difference will be calculated for individuals with HIV-RNA ≥50 cp/mL using the Mantel-Haenszel approach stratified for the stratification factor study site, and non-inferiority will be declared if the corresponding upper one-sided 97.5% confidence limit will be below the margin of 8%. In addition to the risk difference, we will also present a risk ratio and the corresponding 95% CI using the Mantel-Haenszel method. In addition, we will perform a hypothetical estimand analysis using inverse probability weighting to account for intercurrent events such as treatment discontinuation for other reasons than virologic failure. One formal interim analysis will be performed after 50% of the patients have completed the week 24 visit. For the interim analysis, we will use the same methods as for the primary outcome to evaluate the proportion of trial participants with an HIV-RNA ≥50 cp/mL at 24.

The analysis of secondary outcomes will be based on the ITT participant set. For binary outcomes, we will compare proportions between the intervention and the current regimen also using the Mantel-Haenszel risk difference and risk ratio as described above. The change in DDI score will be summarised using median values and quartiles, and differences between the treatment groups will be compared using the non-parametric van Elteren test stratified for the study site. Changes in treatment satisfaction (HIV Treatment Satisfaction Questionnaire (HIVTSQ), HIV Treatment Satisfaction Questionnaire change version (HIVTSQc)), quality of life (World Health Organization Quality of Life Brief Version (WHOQOL-HIV BREF)), mental health (Patient Health Questionnaire) and intact proviral HIV-DNA levels from baseline to week 48 will be assessed in a mixed-effects linear model adjusted for the baseline value as a fixed effect (if applicable) and site as a random effect. Cost data will be evaluated using a generalised mixed-effects linear model.

If we can establish non-inferiority for the primary outcome, we will also test the main secondary outcomes for superiority in a sequential manner: change in DDI score, followed by treatment satisfaction (HIVTSQc). Both secondary outcomes will be tested at a two-sided alpha level of 0.05. This hierarchical gate-keeping procedure keeps the overall type I error rate at 0.05.

### Qualitative analyses

Audio-recorded data from the interviews will be transcribed verbatim and analysed using Thematic Analysis following Braun and Clarke’s approach. Researchers will first familiarise themselves with the data through repeated reading of the transcripts. As interviews are being undertaken (and transcribed) in two languages (French and German), coding will be performed in English on vernacular transcripts to create an overall codebook in close coordination between the teams using MAXQDA software. Codes will be assigned inductively, without a predefined coding framework. The process of identifying themes from codes will involve collaborative sessions among researchers from the two language teams, as initial and final themes will be identified across the complete dataset.

### Independent Data Monitoring Committee

An Independent Data Monitoring Committee (IDMC) will monitor the trial. The IDMC will meet once before the start of the trial and once after 50% of the study participants have completed week 24. The IDMC members will be provided with a safety event report every 6 months. Based on the reports, safety IDMC meetings can be called on request by the sponsor or any of the IDMC member. We will convene an IDMC meeting if >2 individuals experienced virological failure and developed new resistance-associated mutations.

### Data collection and management

All data are collected electronically using a dedicated electronic data capturing system (REDCap) hosted by the Department of Clinical Research of the University of Bern. Only the system administrators have direct access to the server. Data edit checks were implemented limiting entries to appropriate, realistic values. Central data monitoring and validation are performed, which includes verifying completeness, plausibility and consistency of the entered data on a regular basis and querying the sites following up on any ambiguity. In addition, on-site monitoring is part of the quality control activities implemented for this study.

### Patient and public involvement

Patients and public representatives have been and will remain involved at all stages throughout the planning and conduct of B-Free. Documents including study protocols, data collection instruments as well as outreach activities have been reviewed by Patient and Public Involvement members of the SHCS. Furthermore, patient representatives were involved in the development of a pretrial survey that was performed among potentially eligible SHCS participants from three centres. The survey aimed (1) to evaluate their willingness to participate in a clinical trial, (2) to adapt the study schedule to a number of visits acceptable for potential study participants and (3) to align the study outcomes with patients’ perception on the importance of ART characteristics.

To engage with patients and the public, we developed a trial website (www.bfree-trial.ch) to provide information about the aim and current status of the study. Potential participants received a flyer containing the main trial information in lay language. Furthermore, we will provide plain and lay summaries of all publications related to B-Free which will be disseminated to patient groups in collaboration with patient representatives. All trial related information are available in English, German, French, Italian and Dutch. In addition, a patient representative is part of the trial scientific committee.

### Ethics and dissemination

#### Ethical considerations

The study was approved by the competent ethics committees in Switzerland (reference number BASEC 2023–01060). Approval by the Swiss regulatory authority (Swissmedic; reference number 701655) has been obtained before the enrolment of the first participant. Approval from the European Medicines Agency, as well as local ethical approval in the Netherlands and France, will be obtained prior to recruiting participants in these countries. Participant’s written informed consent is obtained by the investigators before enrolment (see consent form in online supplemental material). All participants and their data are handled according to the ethical principles of the Declaration of Helsinki, the respective country-specific law on human research as well as data protection law. This study complies with all applicable standards of the International Council on Harmonization E6 Guideline for Good Clinical Practice (ICH-E6 [GCP] 1996) guideline. The ethics committees and regulatory authorities receive annual safety reports and will be informed about the study stop/end in agreement with local requirements. The trial is registered on clinicaltrials.gov (NCT06037564, registered on 07 September 2023) and in the Swiss National Clinical Trials Portal (SNCTP000005686, registered on 06 November 2023, see online supplemental table S2).

### Publication and dissemination policy

The results of all major B-Free study outcomes will be submitted to peer-reviewed journals that enable Open Access publication. Statistical codes will be made available through a public repository on www.github.com. Data will be deposited in the Bern Open Repository and Information System. All items will be stored with a unique Digital Object Identifier that can be referenced in respective publications.

## Discussion

The B-Free multistage trial is a unique platform to study treatment simplification strategies among PWH with prior virologic failure. The study will fill an important research gap, as the concerns of individuals with multidrug-resistant HIV are currently understudied, and evidence-based treatment recommendations are lacking. Furthermore, given the increasing proportion of PWH who are confronted with comorbidities, our results will offer evidence for ART strategies with a reduced risk of DDI with co-medications for this ageing population. The multistage design embedded within well-described cohorts will allow a continuous and resource-effective evaluation of newly available ART combinations, thereby offering simplified treatment options to most individuals in this important population.

Our approach has several strengths: as the study population is recruited within established cohorts of PWH, participant identification and enrolment are greatly facilitated. In addition, long-term outcome assessment after the end of the study is guaranteed since individuals are prospectively followed within their cohort. Furthermore, the inclusion of study participants from multiple countries and across a variety of study centre types (university, regional hospitals and private physicians) will increase the generalisability of the study results. Finally, the strong collaboration with patient representatives allowed us to design the study according to the priorities and perceptions of PWH, and the integration of a strong qualitative part into our work will allow us to shape and optimise our multistage trial prospectively.

The main limitation of the study is the inability to blind both participants and trial physicians due to the heterogeneous regimens used in the control arm. Nevertheless, laboratory assessment of the primary outcome (HIV viral load at week 48) and the statistical analyses are performed by individuals who are blinded to the treatment allocation.

The B-Free trial takes into account contemporary developments in medical conditions of PWH and addresses some of the most important challenges related to delivering HIV care to an ageing population, while ensuring people’s treatment satisfaction and quality of life. The trial results will provide evidence-based guidance for choosing the optimal treatment strategy for the understudied population of PWH and a history of virological failure.

### Current status of the B-free trial

The B-Free trial study setup was initiated in October 2022, and recruitment started in Switzerland on 13 November 2023. The estimated time of recruitment is 2 years.

## supplementary material

10.1136/bmjopen-2024-094912 online supplemental file 1
