# Supplementary material for: Booster-free anti-retroviral therapy for persons living with HIV and multidrug resistance (B-Free): protocol for a multicentre, multistage, randomised, controlled, non-inferiority trial
Source: BMJ Open. 2024 Nov 21;14(11):e094912. doi: 10.1136/bmjopen-2024-094912 (PMC11590791; doi:10.1136/bmjopen-2024-094912)
Supplement: online supplemental file 1 [file bmjopen-14-11-s001.pdf]

Supplementary Material

Table S1: B-Free trial assessment schedule (stage 1)

Study Periods	Screening	Baseline	Intervention Period				End of Study	Safety Follow-up
Visit	0	1	2	3	4	5	6	7
Time point	-4 weeks	Day 0	Week 4	Week 12	Week 24	Week 36	Week 48	Week 52[4]
STUDY PROCEDURES								
Assess eligibility criteria	x	x						
Obtain written informed consent	x							
Randomization		x						
Administration of study drug		x	x	x	x	x	x	
STUDY ASSESSMENTS WITHIN THE COHORT ROUTINE								
Behavioural data		x			x		x	
Adherence assessment questionnaire		x	x[5]	x[5]	x	x[5]	x	
Routine laboratory tests		x			x		x	x
CD4 count		x			x		x	
STUDY SPECIFIC ASSESSMENTS								
Demographics	x							
Medical history		x						
Documentation of virologic failure and historical ART resistance testing	x							
Concomitant medications	x	x	x	x	x	x	x	
Pill count		x	x	x	x	x	x	
Physical examination	x	x	x[6]	x[6]	x	x[6]	x	
Vital signs	x	x	x		x		x	
Body weight		x			x		x	
Assessment for central nervous system (CNS) events		x	x	x	x	x	x	x
Assessment for treatment limitations due to DDI		x		x	x	x	x	
Assessment of treatment satisfaction (HIVTSQ)		x					x	
Assessment of quality of life (WHOQOL-HIV BREF)		x					x	
Assessment of mental health (PHQ-9)		x					x	
In-depth interviews[1]		x					x	

Table S1 Continued

Study Periods	Screening	Baseline	Intervention Period				End of Study	Safety Follow-up
Visit	0	1	2	3	4	5	6	7
Time point	-4 weeks	Day 0	Week 4	Week 12	Week 24	Week 36	Week 48	Week 52[4]
LABORATORY ASSESSMENTS								
Blood pregnancy test[2]	x							
HBsAg, anti-HBc, anti-HBs	x							
HIV viral load	x	x	x	x	x	x	x	x
HBV viral load[3]		x			x		x	
Safety laboratory tests	x		x	x		x		
Plasma sample storage		x	x	x	x	x	x	
PBMC storage		x					x	
Drug plasma concentration			At any time point if confirmed HIV RNA ≥200 cp/mL					
Genotypic resistance testing			At any time point if confirmed HIV RNA ≥200 cp/mL					

[1] Only in a subset of 60 individuals. Interviews are conducted at baseline and week 48 in 15 individuals from the intervention arm and 15 from the control arm, once in 15 individuals who were ineligible for the trial, and once in 15 eligible individuals who refused to participate in the trial. Please refer to the section on the **"qualitative sub-study"** for more information.
[2] Only done in women of childbearing potential.
[3] Only individuals with a positive anti-HBc and a negative anti-HBs serology ("anti-HBC alone").
[4] This visit will only be performed in individuals with ongoing CNS/ serious adverse events, in participants with ongoing lab abnormalities, or a detectable HIV viral load at week 48. The visit can be done via telephone if no lab testing is needed.
[5] If a pill count cannot be performed (e.g. the participant forgot to return the used medication bottles), they are asked to complete an adherence assessment questionnaire covering the past 4 weeks.
[6] If clinically indicated, based on the judgment of the investigator.

DDI = drug-drug interaction, **HIVTSQ** = HIV Treatment Satisfaction Questionnaire, **WHOQOL-BREF** = Abbreviated World Health Organization Quality of Life questionnaire, **PHQ-9** = Patient Health Questionnaire-9, **HBsAg** = hepatitis B surface antigen, **anti-HBc** = hepatitis B core antibody, **anti-HBs** = hepatitis B surface antibody, **HBV** = hepatitis B virus, **PBMC** = peripheral blood mononuclear cells.

Table S2: Trial registration data

DATA CATEGORY	INFORMATION
Primary registries, trial identifying numbers, and date of registration	clinicaltrials.gov (NCT06037564, 07.11.2023) and Swiss National Clinical Trials Portal (SNCTP000005686, 06.11.2023)
Source of monetary support	Swiss National Science Foundation,) grant number 205829
Sponsor	Insel Gruppe AG, Bern University Hospital, Bern, Switzerland
Sponsor representative	Prof. Dr. med. Gilles Wandeler
Contact for public queries	Prof. Dr. med. Gilles Wandeler, gilles.wandeler@insel.ch
Contact for scientific queries	Prof. Dr. med. Gilles Wandeler, gilles.wandeler@insel.ch
Public title	B-Free Multistage Trial
Official title	Booster-free antiretroviral therapy for persons living with HIV and multidrug resistance (B-Free): A Multicenter, multi-stage, randomized, controlled non-inferiority trial
Countries of recruitment	Switzerland, the Netherlands, France
Health conditions studied	HIV; Drug resistance; Drug-drug interaction
Interventions	Intervention: Doravirine 100 mg administered once daily in combination with co-formulated dolutegravir/lamivudine 50/300
	Control: Continuation of participant's fully suppressive booster-containing antiretroviral therapy at baseline.
Key inclusion criteria	Informed consent as documented by signature.
	Age ≥18 years.
	Documented HIV-1 infection.
	On ART including a pharmacological booster (ritonavir or cobicistat) and at least 2 drugs from classes other than NRTI.
	A history of ART change due to virologic failure.
	HIV-RNA <50 cp/mL at screening and for at least 24 weeks before screening (one blip with less than 200 cp/mL is allowed).
Key exclusion criteria	Creatinine clearance <30mL/min.
	Known hypersensitivity, allergy, or intolerance to DOR, DTG, or 3TC.
	Presence of major drug resistance mutations against DTG (G118R, G140R, Q148H, Q148K, Q148R, R263K) or DOR (V106A, Y188L, F227C, F227L, M230L, Y318F) according to IAS-USA in individual cumulative resistance analyses*.
	Concomitant use of drugs that decrease DTG or DOR blood concentrations.
	Chronic hepatitis B infection.
	Women who are pregnant or breastfeeding.
	Concurrent participation in another ART intervention study.
Study type	Interventional
	Phase 4
	Allocation: Randomized
	Interventional Model: Parrallel Assignment
	Masking: Single (Outcomes Assessor)
	Primary Purpose: Treatment
Date of first enrollment	13 November 2023
Target sample size	210
Recruitment status	Recruiting
Primary outcome	Loss of viral suppression at week 48
Key secondary outcomes	Changes in the burden of drug-drug interactions (DDI) from week 0 to 48.
	Changes in treatment satisfaction between weeks 0 and 48.

Supplementary Material – Patient informed consent

Request for participation in medical research, "B-Free"

Study title: **Booster-free antiretroviral therapy for persons living with HIV and multidrug resistance: A multicentre multi-stage randomized trial ("B-Free")**

Layperson title: **Booster-free HIV therapy for persons with pre-existing HIV resistance**

Dear Madam or Sir

We would like to inform you about the study mentioned above and ask you whether you would like to participate. Before the study treatment consisting of the new combination of Dovato® and Pifeltro® may be used, its efficacy must be determined.

In this study, we want to evaluate if the booster-free study medication Dovato® and Pifeltro® is as effective as your previous HIV treatment that includes a booster. A booster does not act directly against the viruses, but rather supports and prolongs the efficacy of the other active substances in the HIV treatment. However, boosters can cause interactions with other medicines not used for the HIV infection. This is why we want to investigate whether the new treatment results in fewer interactions between the HIV treatment and other drugs, and whether your quality of life improves with the study treatment.

Your participation is voluntary. The following **patient information** should help you decide. You can **discuss** any questions about study participation **with the study doctor**. The study doctors are the ones who are responsible for the study and who look after you during this time. If you want to take part, please sign the **informed consent form** at the end. With your signature, you confirm that you have read and understood the patient information. If there is something you do not understand, please ask the study doctor.

The patient information and informed consent form consist of four parts:

Part 1 **The most important points at a glance**
Part 2 **More details: Information on the study**
Part 3 **Data protection and insurance coverage**
Part 4 **Informed consent form**

In **Part 1** you will get an overview of the study. In **Part 2** we explain the study procedures and the background of the study in more detail. **Part 3** contains the information on data protection and insurance coverage. With your signature at the end of the document, **Part 4**, you confirm that you have understood everything and that you consent to take part.

This study will be conducted by Inselgruppe. The responsible person is Prof. Dr. med. Gilles Wandeler, Senior Physician at the Department of Infectious Diseases at the Inselspital in Bern, Switzerland. The study is financed by the Swiss National Science Foundation (SNSF).

As part of this study, the following person is responsible for you:

Name	Dr. med. Bernard Surial
Address	Inselspital, Department of Infectious Diseases
Telephone	+41 (0)31 632 88 44 → Weekdays 8:00am-5:00pm<br>+41 (0)31 632 21 11 → Ask for Infectious Diseases doctor on call (24-hour availability)
E-mail	b-free@insel.ch

Part 1:
The most important points at a glance

1. Why are we doing this study?

In this study, we are investigating whether the study medication Dovato® and Pifeltro® is as effective as previous tried-and-tested HIV treatments. The combination of Dovato® and Pifeltro® is well suited for patients with resistance to available HIV medication and previous treatment failure.

The study medication is considered to be effective if the HIV viral load can be suppressed with this treatment. Furthermore, we would like to assess whether the study medication results in fewer interactions with other drugs, and if your quality of life improves with this medication. In **Chapter 4** you will find out more about the scientific background of the study.

2. What do you have to do if you take part?

Your participation in this study will last 52 weeks. We will invite you for 8 study visits. At least 3 of these visits are part of your general treatment, and will take place regardless of your participation in the study. The other visits are additional study visits. A visit will last between 30 minutes and 1 hour and 30 minutes. The number of visits is stated in the **table in Chapter 5**.

If you decide to participate, you will be allocated randomly to one of two groups: the trial or the control group. In the trial group, you will receive the study medication Dovato® and Pifeltro®, 2 tablets to be taken at the same time once daily. In the control group, you will continue with your current HIV treatment.

In addition, we would like to conduct a 60 to 90-minute interview with 30 study participants from the different study sites at two different time points. These interviews will be conducted according to a guideline and will be recorded for the scientific analysis of the statements. The interviews will be conducted by specialists from the Universities of Bern and Lausanne. With these interviews, we aim to better understand patient needs in terms of clinical studies and research in general, and to analyse the expectations for optimal HIV treatments. The analyses of these interviews will be included in the final results of this study.

In **Chapter 5** you will find out more about the course and procedure of the study.

3. What are the benefits and risks associated with participation?

Benefits

You might have no direct benefit by participating in the study. However, you may help future patients by taking part. A benefit arises from the fact that we will gain further insights into the impact of booster-free therapeutic approaches, which may have the potential of having fewer side effects compared to current HIV treatments.

Risks

The drugs Dovato® and Pifeltro® are approved in Switzerland, and are prescribed on a regular basis for the treatment of HIV. However, there are insufficient studies on the administration of this combination of Dovato® and Pifeltro® to patients with a history of HIV resistance and virological failure, and therefore Dovato® and Pifeltro® are not licenced for this group of patients. Their efficacy for your situation remains unknown.

In **Chapter 6** you will find further information on the risks and burdens.

INSELSPITAL
UNIVERSITÄTSSPITAL BERN
HÔPITAL UNIVERSITAIRE DE BERNE

Part 2:
More details: Information on the study

4. The scientific background of the study

4.1 Background: Why are we doing this study?

Effective antiretroviral therapy has increased the life expectancy of people with HIV so that it almost corresponds to that of people without HIV. As we get older, concomitant diseases such as cardiovascular diseases become more frequent, which may require additional medication. Therefore, interactions between HIV drugs and concomitant medications represent an increasing problem. In the Swiss HIV Cohort Study (SHCS), for example, two thirds of the people aged 50 years or older have one or more other diseases, for which almost 50% have to take one or more additional medication.

People who previously had HIV treatment failure usually receive what is known as a "boosted" HIV treatment, which contains the ingredients ritonavir or cobicistat. These substances are called "boosters". Boosted HIV treatments are particularly prone to lead to drug interactions. In the SHCS, three quarters of the drug interactions occurred in patients who received boosted HIV treatments. These drug interactions may result in serious complications, and sometimes affect the treatment of concomitant diseases, e.g. with chemotherapy or blood-thinning medication.

In this study we want to investigate the efficacy and acceptance of booster-free HIV treatment in treatment-experienced individuals. The high efficacy and good tolerability of Dovato® and Pifeltro® make this combination a suitable therapy, with the objective of simultaneously reducing the risk of drug interactions. The drugs Dovato® and Pifeltro® are approved in Switzerland, and are prescribed on a regular basis for the treatment of HIV. However, there are insufficient studies on the administration of this combination to patients with HIV resistance and a history of virological failure and therefore Dovato® and Pifeltro® are not licenced for this group of patients.

Furthermore, we would like understand the needs of people with HIV in terms of HIV research, to know how the current study is evaluated, and what the expectations of future HIV treatments are. For this, in-depth interviews will be conducted with a selection of 30 patients from the different study sites, and these will then be analysed by experts from the universities of Bern and Lausanne. You can take part in the study even if you do not want to do these interviews.

4.2 Design of the study: How will we proceed?

Participants will be allocated randomly to groups in our study. This is important to have reliable study results. In our study, there are two groups at a 1:1 ratio:

- **Group 1** (trial group) receives the study medication (Dovato® and Pifeltro®), one tablet of each daily. The tablets can be taken independently of meals.
- **Group** 2 (control group) continues the current HIV treatment with booster.

Both you and the study doctor as well as the study staff will be aware of what treatment you will receive.

4.3 Regulations for scientific research in humans

We are conducting this study in accordance with Swiss laws and all internationally recognised guidelines. The competent Ethics Committee and Swissmedic have reviewed and approved the study.

This is an international study conducted in Switzerland and additionally in European countries. In total, we will include 210 study participants, of whom 120 will be recruited in Switzerland.

You can also find a description of this study on the website of the Swiss Federal Office of Public Health: www.kofam.ch using the SNCTP registry number 000005686 or the BASEC number 2023-01060.

5. Course of the study

5.1 What do you have to do if you participate in the study?

Participation in the study is voluntary and lasts 52 weeks. You must follow the schedule (→ Chapter 5.2) and all requirements set by your study doctor.

You will need to tell your study doctor

- if your health condition changes, e.g. if you feel worse or if you have new symptoms; this also applies if you end the study early (→ Chapter 5.3 and 5.4)

You should also be aware of the following:

- During participation, women of childbearing age must use contraception to avoid pregnancy (→ Chapter 5.5).

5.2 What happens at the visits?

Over the course of your participation, you will come in for 8 study visits. At least 3 of these visits are part of your general treatment and also take place regardless of your participation in the study. The other visits are additional visits and are only part of the research study. A visit lasts about 30 minutes to 1 hour and 30 minutes. The sequence of the visits is outlined in the table below.

We will do the following at all of the visits:

- We will answer your questions.
- We will check the regularity of your medicine intake. This is why you must always bring all HIV medication with you.
- We will give you the study medication/HIV medication.
- We will do a physical examination.
- We will assess any new physical symptoms.
- We will record any new medicines you are taking.
- We will take blood samples, namely at least 14.5 mL (approximately 3 teaspoons) and max. 31 mL (approximately 6 teaspoons).

At individual visits, we will also do the following:

- At the first visit, we will record your medical history and, if you are a woman of childbearing potential, a pregnancy test will be performed.
- We measure your weight, blood pressure and pulse.
- You will receive questionnaires about your quality of life and mental health.
- We will ask you questions regarding employment, physical activity, mental well-being, alcohol and drug consumption, as well as relationships and sexual behaviour.
- We will perform urine tests, and these will require a urine sample from you (maximum 7.5 mL, or approximately 1.5 teaspoons)

These examinations enable us to see whether the study treatment works and whether it is safe, whether you have fewer interactions with other medicines and whether your quality of life has changed.

We will arrange the visits together with you.

The schedule on the next page shows all of the visits in detail. The visits and activities highlighted in grey are specific for the B-Free study.

Schedule: General and additional examinations (activities in grey are study specific)

Study visit/ visit	0	1	2	3	4	5	6	7
	Screening	Day 0	Week 4	Week 12	Week 24	Week 36	Week 48	Week 52
Duration	1 hr 30 min	1 hr 30 min	30 min	30 min	1 hr 30 min	30 min	1 hr 30 min	15 min
Checking in- and exclusion criteria for the study	✓	✓						
Signing patient information and informed consent form	✓							
Random allocation to trial or control group		✓						
Recording medical history, general and HIV-specific		✓						
Recording demographic data	✓							
Pregnancy test for women of childbearing potential	✓							
Various questionnaires		✓			✓		✓	
Blood + urine sampling	✓	✓	✓	✓	✓	✓	✓	✓
Measurement of blood pressure and pulse	✓	✓	✓		✓		✓	
Body weight measurement		✓			✓		✓	
Physical examination	✓	✓	(✓)	(✓)	✓	(✓)	✓	
Recording of all current medication	✓	✓	✓	✓	✓	✓	✓	
We will ask your doctor whether there were any problems with other drugs due to the study medication		✓		✓	✓	✓	✓	
Evaluate for symptoms		✓	✓	✓	✓	✓	✓	✓
Assess study medication adherence		✓	✓	✓	✓	✓	✓	
Handing out study medication		✓	✓	✓	✓	✓	✓	
In-depth interview, if agreed (approx. 60-90 min)		✓					✓	

5.3 When does the study participation end?

Your participation will last 52 weeks and ends after the eighth visit. You can also end your participation earlier at any time (→ Chapter 5.4). You do not need to explain why you no longer want to participate. If you decide discontinue the study early, please talk to your study doctor.

Even if you discontinue prior to the end of the study, we will continue to treat you and look after you as good as medically possible according to the current standards (→ Chapter 5.4 for alternative treatment options). For your safety, we will examine you at the time of study discontinuation.

If you end the study early, we ask that you continue to inform your study doctor if your health condition changes, e.g. if you feel worse or have new symptoms. In case you stop the study early, we will analyse the data and samples collected until that point (e.g. blood values) for the study.

We might also have to ask you to end the study early. This could happen, for example, if you experience serious side effects occur due to the study medication, or if you develop new HIV resistances during the study.

5.4 What happens if you do not wish to take part?

Even if you do not take part in this study, we will treat you and look after you with the best possible medical care according to the current standards. If you do not wish to take part in the study, your study doctor will advise you of alternative treatment choices.

5.5 Pregnancy

There may be pregnancy risks. It is not known whether the study medication may harm an unborn or breastfed child. If you are pregnant, want to become pregnant, or are breastfeeding, you cannot take part in the study.

For women of childbearing age, a pregnancy test will be performed before the start of the study. There are still not enough data as to whether the study medication has an effect on the unborn child. This is why study participants must use a method of contraception during the study (condoms, hormonal methods such the pill, or intrauterine device). Your study doctor will discuss the suitable methods with you.

If you become pregnant despite contraception during the study, you have to inform your study doctor immediately. In this case, there will be regular follow-up of your pregnancy, and your study doctor will collect health data about your child. Your study doctor will talk to you about your HIV treatment options, which may also include further intake of the study medication. To continue the study medication Dovato® and Pifeltro®, you must sign a separate informed consent form (see *information and informed consent form for pregnancy follow-up in pregnant study participants*). Your study doctor will discuss with you what happens next.

6. Risks, burdens and side effects

6.1 What risks and burdens may occur?

As with any medical treatment, there are risks and burdens involved in participation in this study. Both Dovato® and Pifeltro® are approved in Switzerland and are prescribed on a regular basis. We already know many of the associated risks, while others may remain unknown. This uncertainty is common in the study setting. You can find a list of the most common and most serious risks in **Chapter 6.2**. Many side effects can be treated. We will inform you of any new finding regarding risks and side effects during the study.

In addition, there are risks involved in the medical examinations that we perform in this study. You will already know some examinations. You can find a list of these risks of the examinations in **Chapter 6.3**.

6.2 The most common and most serious risks caused by the study medication

You can find information here about the most common side effects that we already know about.

Dovato® film-coated tablets 50 mg/300 mg (active ingredients: dolutegravir and lamivudine)

Very common side effects (occurs in more than 10%): Headache, diarrhoea, nausea

More information can be found in the medical product information.

Pifeltro® film-coated tablets 100 mg (active ingredient: doravirine)

Very common side effects (occurs in more than 10%): none.

More information can be found in the medical product information.

6.3 Risks and burdens caused by examinations in the study

We will carry out various medical examinations for this study (→ Chapter 5.2). These examinations are tried-and-tested procedures. Nevertheless, they may involve risks and burdens, which means that they may be unpleasant or have unwanted side effects. In this study, blood sampling may cause bruising, bleeding or swelling at the puncture site. It may rarely cause an infection at the puncture site.

7. Financing and compensation

This study is organised by the sponsor and wholly financed by the Swiss National Science Foundation. The study medication and HIV medication of the control group as well as the routine medical checks are covered by your health insurance company. Study visits 2, 3, 5 and 7 (in accordance with the schedule) will be conducted as part of the study and will not be charged to the health insurance company.

The researchers involved do not have a financial benefit from the conduct of this study.

There will be no additional costs for you or for your health insurance company from your participation in the study. For study visits 2, 3 and 5 (in accordance with the schedule) you will receive a compensation of 50 Swiss Francs for each visit as well as reimbursement of the travel costs. Participation in the in-depth interviews will be compensated with an additional 50 Swiss Francs per interview.

8. Results from the study

Your study doctor will inform you of results that are important to you. There are also incidental findings. Incidental findings are "side results" that are not anticipated. This can be, for example, results from liver values. We will inform you if these incidental results are important for your health.

We will inform you, for example, if we discover a disease by chance that you know nothing about and that we can treat.

There are also the overall results of the study that come from the data of all participants. This includes, e.g. the fact that we know more about the efficacy and drug interactions (→ Chapter 4.1). Your study doctor will be happy to give you a summary of the overall study results at the end of the study if you wish.

Part 3:
Data protection and insurance coverage

9. Protection of data and samples

We protect your data (e.g. information such as blood pressure and pulse from your medical history), and your samples (e.g. your blood samples). Switzerland has strict legal regulations in place for the protection of data and samples.

9.1 Coding of data and samples

As with all studies, information arises from the examinations (e.g. blood values, blood pressure, pulse). These data will be entered in an electronic database. The data will be documented in coded form. "Coded" means that your data will not be connected with personal information such as name, date of birth or place of residence, but rather only via a code. We manage a list at the institution that we use to determine which code belongs to you. This means that your name, your date of birth or your place of residence, etc. are *not* directly written in the database. This list will remain with us at the institution for 10 years. Nobody else will receive this list.

At the end of the study (at the earliest time point after the legally stipulated storage duration) your data will be completely anonymised. This means that it will no longer be possible to identify you without unreasonable effort. Various measures are used for anonymisation, e.g. destruction of the code and the list.

All samples that are analysed in external laboratories (e.g. blood and urine samples) are always coded in the way described above. Your personal data are therefore protected if we send samples.

9.2 Secure processing of data and samples during the study

The sponsor is responsible for the secure processing of your data and samples from this study. The sponsor is responsible for the compliance with the applicable laws, such as data protection laws

In this study, your data will be recorded in an electronic database of the sponsor. The data are stored on a server in Switzerland, and the access rights are restricted to a group of people who need access for work on the study. Nevertheless, there is always a residual risk that third parties can access your personal data (e.g. risk of hacking).

It is often important for your GP to share data from your medical history with the study doctor. This also applies to other doctors treating you. By giving your consent at the end of the document, you give your permission for this.

During the screening visit, we will test you for hepatitis B virus. In the event of a new positive hepatitis B virus result, certain information (including name, sex, date of birth and place of residence) will be reported in uncoded form to the cantonal physician and the federal office of public health (FOPH) to comply with national health regulations.

9.3 Secure processing of data and samples after the study

The sponsor remains responsible for the secure processing of your data and samples even after the end of the study. The law mandates that all study documents, including the data in the database, are stored for at least 10 years.

If, after the end of the study, there are still residual samples left, we will collect them and store them coded in a safe place after the end of the study. In this way, they can be used later for further analyses (→ Chapter 9.4). This type of collection of coded samples is called a "biobank". There are strict rules for biobanks to make sure the information from your samples is well protected.

After completion of a study, the results are usually published in scientific journals. For this, the results are assessed by other specialists. Your coded data must therefore be forwarded to these specialists. However, the data must not be used for new research purposes. This would require your separate informed consent (see 9.4).

9.4 Further use of your data and samples in other future studies

Your data and samples from this study are very important for future research. Data and samples that were not completely used for this study may possibly be used for other studies. We need your separate informed consent for the further use of your data and your samples. This is voluntary. Please read the additional informed consent form at the end of the document carefully. Please sign the informed consent form if you would like to support further research in the future with your data and samples. Even if you do not consent to this, you can still participate in the study.

9.5 Rights of inspection during controls

The conduct of this study may be reviewed. The review is carried out by authorities such as the competent Ethics Committee or the regulatory authority Swissmedic or also by foreign regulatory authorities. The sponsor also has to carry out such reviews to ensure the quality of this study and the results.

For this, a few specially trained individuals will take a look at your personal data and medical history. The data are therefore *not* coded for this review. The individuals who see your uncoded data are bound by medical confidentiality.

10. Insurance coverage

You are insured if you sustain an injury due to the study. The procedure is regulated by law. The sponsor has obtained an insurance policy for this from Zürich Versicherungs-Gesellschaft AG in Zurich, Switzerland. If you think you have sustained an injury due to the study, please contact your study doctor or the insurance company directly.

Part 4: Informed consent forms

This informed consent has two informed consent forms:

- Informed consent form for participation in this study B-Free
- Informed consent form for the further use of data and samples from this study in coded form (only if you agree to take part in B-Free)

Please read the forms carefully. Please ask us if there is something you do not understand or if there is something you would like to know. Your written consent is required for participation.

Informed consent form for participation in the study B-free

**BASEC number**	2023-01060
**Study title**	Booster-free antiretroviral therapy for persons living with HIV and multidrug resistance: A multi-centre multi-stage randomized trial ("B-Free")
**Layperson title**	Booster-free HIV therapy for persons with pre-existing HIV resistance
**Responsible institution**<br>(Sponsor with address)	Inselgruppe AG, Freiburgstrasse 8, 3010 Bern<br>Represented by Prof. Dr. med. Gilles Wandeler Inselspital, Department of Infectious Diseases
**Site where the study is to be conducted**	Inselspital, Department of Infectious Diseases
**Head of the study at the study site**	Dr. med. Bernard Surial
**Participant**<br>Last name and first name (please print):<br>Date of birth:	

- I have received verbal and written information about the study from the study doctor whose signature appears below.
- The study doctor has explained the purpose, the course and the risks of the study.
- I am taking part in the study of my own free will.
- The study doctor explained to me what possible standard treatments are available outside of the study.
- I have had enough time to make my decision. I can keep the written information, and I will receive a copy of my written informed consent form.
- I am free at any time to stop taking part. I do not have to explain why. Even if I stop taking part, I will still receive my medical treatment. The data and samples collected until then will continue to be stored and evaluated as part of the study.
- If it is better for my health, the study doctor may exclude me from the study at any time.
- I have understood that my data and samples will only be passed on in coded form. The sponsor guarantees that data protection in accordance with Swiss standards will be observed.
- In the event of results and/or incidental findings that affect my health directly, I will be informed.
- My GP may share data from my medical history that are important for the study with the study doctor. This also applies to other doctors treating me.
- The competent specialists employed by the sponsor, the Ethics Committee and the medicinal product authority Swissmedic may view my uncoded data for inspection purposes. All of these individuals are bound by medical confidentiality.
- I know that the sponsor has taken out an insurance policy. This insurance policy pays if I sustain an injury – but only if the injury is directly associated with the study.

- **Consent for two in-depth interviews (each 60 to 90 minutes long):**
  ☐ YES ☐ NO

Place, date	Signature of the participant

Confirmation of the study doctor: I hereby confirm that I have informed this participant of the nature, significance and scope of the study. I affirm that I shall fulfil all of my obligations in connection with this study in accordance with Swiss law. If at any point during the conduct of the study I learn of aspects that could influence the willingness of the participant to take part in the study, I will inform him/her of this immediately.

Place, date	Last name and first name of the study doctor (please print)<br><br><br>Signature of the study doctor

Informed consent form for the further use of data and samples in coded form

This informed consent does not affect your personal participation in a study. "Further use" means that data and samples can be stored beyond the time of your study participation and used in coded form for further research. This may mean, for example, that a blood sample and corresponding laboratory values from you are statistically analysed together with a large number of other values or new examinations are carried out on them.

**BASEC number:**	2023-01060
**Study title:**	Booster-free antiretroviral therapy for persons living with HIV and multidrug resistance: A multi-centre multi-stage randomized trial (“B-Free”)
**Layperson title:**	Booster-free HIV therapy for persons with pre-existing HIV resistance
**Participant:**<br>Last name and first name (please print):<br>Date of birth:	

- I consent to my coded data and samples from this study being used for further medical research. The samples will be stored in a biobank. They will then be available for an indefinite period of time for other future research projects.

- I have understood that the samples are coded and the key will be stored securely.

- The data may be analysed here and abroad and stored in a database here or abroad. The samples may be examined here or abroad and stored in a biobank. Research institutions abroad must observe the same data protection standards as applicable in Switzerland.

- I am making this decision of my own free will, and I can withdraw this decision at any time. If I withdraw, all of my data will be anonymised and my samples will be destroyed. I only have to inform my study doctor and do not need to justify this decision.
- Normally, all data and samples will be assessed as a whole. If a result randomly appears that is very important for my health, I will be contacted.

Place, date	Signature of the participant

Confirmation of the study doctor: I confirm that I have informed the participant of the nature, significance and scope of the further use of samples and data.

Place, date	Last name and first name of the study doctor (please print)<br><br>Signature of the study doctor
