# Supplementary material for: Booster-free anti-retroviral therapy for persons living with HIV and multidrug resistance (B-Free): protocol for a multicentre, multistage, randomised, controlled, non-inferiority trial
Source: BMJ Open. 2024 Nov 21;14(11):e094912. doi: 10.1136/bmjopen-2024-094912 (PMC11590791; doi:10.1136/bmjopen-2024-094912)
Supplement: Supplementary file 2 [file bmjopen-2024-094912.reviewer_comments.pdf]

PEER REVIEW HISTORY

BMJ Open publishes all reviews undertaken for accepted manuscripts. Reviewers are asked to complete a checklist review form and are provided with free text boxes to elaborate on their assessment. These free text comments are reproduced below.

ARTICLE DETAILS

Title (Provisional)

Booster-free antiretroviral therapy for persons living with HIV and multidrug resistance (B-Free): protocol for a multicenter, multi-stage, randomized, controlled, non-inferiority trial

Authors

Surial, Bernard; Ballif, Marie; Braun, Dominique; Calmy, Alexandra; Bernasconi, Enos; cavassini, matthias; Tissot, Frédéric; Stockle, Marcel; Schmid, Patrick; Fux, Christoph A.; Van der Valk, Marc; Brinkman, Kees; Mudrikova, Tania; Bonnet, Fabrice; Leleux, Olivier; Saúde, Manuela; Hirter, Daniela; Schwab, Nathalie; Limacher, Andreas; Rintelen, Felix; Kouyos, Roger D.; Haerry, David; Zambrano Ramos, Sofia Carolina; Egloff, Martina; Akré, Christina; Peytremann-Bridevaux, Isabelle; Rauch, Andri; Wandeler, Gilles

---

This article was not externally reviewed at BMJ Open. Protocols that have been independently assessed prior to submission to BMJ Open are usually fast-tracked to publication on the grounds that further substantial changes will not be possible. This independent assessment will usually be external, independent review for both a substantial grant award from a non-commercial or government funder and ethics approval.
